# To the Proprietors of the London Medical and Physical Journal

**Published:** 1815-07

**Authors:** John C. Yeatman

**Affiliations:** of the Royal College of Surgeons, &c. To Mr. Souter, 1, Paternoster-row, London.


THE LONDON

### Medical and Physical Journal.

1 OF VOL. XXXIV.] JULY, 1815. [NO. 197.

*THE following Letter was accompanied with a permission to publish it, and requires no commentary. It is written with an ingenuous warmth, which does honour to the writer, and with sentiments of indignation, which we are pleased to see excited in so respectable a quarter. We know not how to express our gratitude to Mr. Yeatman, and most sincerely hope his example will be imitated by every gentleman who is made acquainted with similar transactions. We have received other communications confirming Mr. Y.'s suspicions, but unaccompanied with any express permission to publish them.*

*Again the Proprietors take this opportunity of offering their sincere acknowledgments for increasing orders; and the Editors feel flattered that their labours are so satisfactory to the enlightened, liberal, and impartial, members of the faculty.*

*To the Proprietors of the London Medical and Physical Journal.*

GENTLEMEN, *Frome, June 6th,* 1815.

THE nefarious practice of denying the existence of one publication, with the view to obtain the sale of another, is extremely abhorrent to an honourable mind. Had the attempt to which I allude been confined to Dr. Vaughan's bookseller, your numerous readers might have supposed it originated in mistake; but, when they find the same attempt made in England as in Wales, they have some reason to fear it will become a matter of serious notoriety.

A surgeon of Trowbridge, in this county, ordered his bookseller, a short time ago, to supply him with your Journals, when another periodical work was palmed upon him, on the ground that your's had been discontinued.

The mass of valuable information in the thirty-three volumes of the "London Medical and Physical Journal," and the able and impartial manner in which it has been conducted from its commencement in 1799 to the present period, entitle it to the protection and support of every member of the faculty. Hence I feel myself called upon (without, however, wishing to state the names of the parties concerned) to apprise you of the above fact.

I am, Gentlemen,

Your very obedient and devoted Servant,

JOHN C. YEATMAN,

*Of the Royal College of Surgeons, &c.*

*To Mr. Souter,*

1, *Paternoster-row, London.*

